# Long Rides

**Published:** 1885-01

**Authors:** 


### LONG RIDES.

Turpin's ride to York is a myth, though it is based on the story told of Nick, who in 1676 is said to have robbed a sailor at Gadshill at 4 o'clock in the morning, and to have ridden a bay blood mare all the way to York, where, after attending to the wants of his steed and himself, he dressed himself in gay clothes, strolled to the Bowling Green, and there meeting the Lord Mayor, asked him the hour. It was 7:45 o'clock. This incident procured for him what old Weller so ardently desired, "a halibi." That one horse could have done the journey in the time was a manifest impossibility. In 1831 Mr. George Osbaldeston wagered a thousand pounds that he would ride 200 miles in 10 hours, and he accomplished the distance in 7 hours 10 minutes and 4 seconds, but he was allowed 28 horses, and he was further allowed 1 hour 22 minutes and 56 seconds for stoppages, and he rode round and round the four-mile course on Newmarket Heath. Cooper Thornhill's ride of 213 miles (April 29, 1745,) along the turnpike road, from Stilton to London, from London to Stilton, and again from Stilton to London, was accomplished with 19 horses in 11 hours, 33 minutes, and 46 seconds, being neariy 19 miles an hour. Mr. Osbaldeston's time was upward of 28 miles an hour. Cooper Thornhill's nineteenth horse was a hunter belonging to the Duke of Ancaster, and he rode it without stopping from the "White Horse" at Wormley. On the following morning, Cooper Thornhill, "quite active and in perfect health," rode back from London to "The Bell" at Stilton.—*Notes and Queries.*

